# Totally laparoscopic versus conventional ileoanal pouch procedure – design of a single-centre, expertise based randomised controlled trial to compare the laparoscopic and conventional surgical approach in patients undergoing primary elective restorative proctocolectomy- LapConPouch-Trial

**DOI:** 10.1186/1471-2482-6-13

**Published:** 2006-11-24

**Authors:** Dalibor Antolovic, Peter Kienle, Hanns-Peter Knaebel, Jan Schmidt, Carsten N Gutt, Jürgen Weitz, Moritz Koch, Markus W Büchler, Christoph M Seiler

**Affiliations:** 1 Department of Surgery, University of Heidelberg, Heidelberg, Germany

## Abstract

**Background:**

Restorative proctocolectomy is increasingly being performed minimal invasively but a totally laparoscopic technique has not yet been compared to the standard open technique in a randomized study.

**Methods/design:**

This is a two armed, single centre, expertise based, preoperatively randomized, patient blinded study. It is designed as a two-group parallel superiority study. Power calculation revealed 80 patients per group in order to recruit the 65 patients to be analysed for the primary endpoint. The primary objective is to investigate intra-operative blood loss and the need for blood transfusions. We hypothesise that intra-operative blood loss and the need for peri-operative blood transfusions are significantly higher in the conventional group. Additionally a set of surgical and non-surgical parameters related to the operation will be analysed as secondary objectives. These will include operative time, complications, postoperative pain, lung function, postoperative length of hospital stay, a cosmetic score and pre-and postoperative quality of life.

**Discussion:**

The trial will answer the question whether there is indeed an advantage in the laparoscopic group in regard to blood loss and the need for blood transfusions. Moreover, it will generate data on the safety and potential advantages and disadvantages of the minimally invasive approach.

## Background

There is little comparative data on restorative proctocolectomy performed via conventional or minimal invasive surgical approaches. The technical feasibility of the laparoscopic approach for performing restorative proctocolectomy has been shown in several series in specialized centres [1-4]. However, there is controversy in the literature on the actual benefit of minimal invasive techniques for extensive colorectal procedures. Numerous smaller randomised and case controlled studies in different fields of colorectal surgery have shown distinct advantages for laparoscopic compared to open procedures in the early postoperative phase [5-8], but the large randomized COST-study on colorectal cancer procedures could only find marginal short term quality of life benefits in the minimally invasively treated group [9]. The majority of studies exclusively using laparoscopically assisted techniques with a Pfannenstiel incision have not been able to show advantages for the laparoscopic procedure [10-12]. Only one smaller case-matched study documented superiority for the laparoscopically assisted group in terms of faster return of intestinal function and shorter hospital stay [1]. Another recently published case matched study found no significant differences in regard to complications and an equivalent quality of life after one year [13]. This is in accord with the only randomized trial comparing hand-assisted laparoscopic to open restorative proctocolectomy [14]. Morbidity was similar in both groups and quality of life in the first three months was equivalent questioning the postulated advantage of the minimal invasive approach in regard to early postoperative recovery.

On the other hand, most studies revealed longer operative times and higher costs for minimal invasive restorative proctocolectomy, a possible drawback in times of reduced allocation of financial resources for health care systems around the world [1,11,13,14]. An increase of intraoperative bleeding and an enhanced need for blood transfusions have been postulated as further disadvantages of minimal invasive techniques in major colorectal surgery [15,16].

Currently the conventional approach to restorative proctocolectomy remains the gold standard; however, specialized centres are increasingly performing this operation laparoscopically. Conventional restorative proctocolectomy has not yet been compared to a totally laparoscopic technique in a prospective randomised study. The only randomized study on minimal invasive restorative proctocolectomy available compared a laparoscopically assisted technique with a Pfannenstiel incision to a standard conventional approach [14]. Major drawback of this study is the use of a laparoscopically assisted technique reducing the potential advantages of the minimal invasive approach compared to a totally laparoscopic approach. The Department of Surgery in Heidelberg has published the largest series of laparoscopically assisted and totally laparoscopic restorative proctocolectomies [4,17]. After having shown the technical feasibility of the totally laparoscopic procedure and having overcome the learning curve in 50 patients, a randomised controlled study with the current laparoscopic technique in comparison to the traditional method is now crucial in order to define the future role of the new approach.

## Methods/design

### Trial population

LapCon-Pouch includes patients over 18 years of age (or over 14 with written consent by their legal guardian) who are planned for an elective restorative proctocolectomy with an ileoanal pouch and are eligible for both surgical approaches, i.e. conventional or totally laparoscopic. Therefore patients with ulcerative colitis or familial polyposis fulfilling the above criteria can be included. Patients having undergone previous abdominal surgery with a large incision are excluded because these patients, if operated laparoscopically, would not profit from the smallest potential advantage, a more favourable cosmetic result. In the first version of the protocol patients with malignancy were explicitly excluded because at that time the safety of laparoscopic procedures was not yet proven. In the meantime several randomized studies have been published that demonstrate the safety of minimal invasive techniques for performing oncologic colorectal surgery also in regard to the long-term oncological outcome. Therefore, the inclusion criteria were expanded in order to allow the inclusion of non-advanced colorectal cancers (≤T2). A detailed overview of all eligibility criteria are given in Table 1.

### Study design and objectives

The LapConPouch-Trial is designed as a pre-operatively randomised, controlled single centre, expertise based trial of patients who undergo primary elective restorative proctocolectomy. It is designed as a two-group parallel superiority study. The primary objective of this study is to compare a totally laparoscopic with a conventional approach for performing restorative proctocolectomy in regard to intraoperative blood loss and need for perioperative transfusions, i.e. within the first 24 hours after surgery. We hypothesise that intraoperative blood loss and the need for perioperative blood transfusions are significantly higher in the conventional group. When analysing the prospectively gathered data of the previous laparoscopically assisted series and the totally laparoscopical series at the Department of Surgery in Heidelberg, major differences between the two groups were the amount of blood loss, the number of blood transfusions and the length of hospital stay. Most of the dissection in the laparoscopically assisted group was done conventionally through the Pfannenstiel incision and the results in this group were indeed comparable to the results of our large group of conventional restorative proctocolectomies. About 35% of patients needed blood transfusions perioperatively. In contrast, none of the first 50 patients undergoing totally laparoscopic restorative proctocolectomy needed any blood transfusions. On the basis of these data, the primary end-point was selected and the power calculation done.

Additionally a set of surgical and non-surgical parameters related to the operation will be analysed. Secondary objectives are the time of procedure in minutes counted from first incision to last suture, postoperative pain measured observer and patient blinded, quantitatively with the VAS (0–100) and the McGill pain questionnaires as well as the amount of analgesic drug used on day two after surgery. Lung function will be assessed preoperatively and on day 2 postoperatively. Quality of life will be measured by the SF-36 questionnaire (validated in German) and body image and cosmesis will be evaluated by a body image questionnaire (non-validated) preoperatively and 3, 6 and 12 months after surgery. Comparison of several time components and frequencies of early and late onset complications as well as ranking for qualitative analysis between both groups will be performed. Last, overall length of postoperative hospital stay in days counting from the day of the primary operation (restorative proctocolectomy) will be documented.

### Interventions

#### General considerations for all patients

Before surgery, all patients undergo a standard mechanical bowel preparation and receive prophylactic broad spectrum antibiotics preoperatively. After induction of anaesthesia, a nasogastric tube and a bladder catheter is inserted. The patients are positioned in a supine modified lithotomy position in padded Allen stirrups. Particular care is taken to preserve the ileocolic vessels in order to optimize perfusion of the pouch. The apex of the pouch needs to reach al least 1 to 2 cm below the symphysis, if not, necessary lengthening manoeuvres will be performed. The root of the mesentery is always mobilized. The pouchanal anastomosis is normally fashioned in a standardized double stapling technique. Generally a circular stapler with a size of 28 or 31 mm, depending on the width of the sphincter, will be used for fashioning the stapled anastomosis. Patients will receive a covering loop ileostomy unless explicitly chosen otherwise by the patient or the treating surgeon. After completion of the pouchanal anastomosis drains are routinely placed in the small pelvis.

#### Conventional approach: standardised technique

A standard median laparotomy from the symphysis to the upper abdomen is done. First, descending, transverse and ascending colon including both flexures are mobilized, generally the greater omentum is left in situ. Dissection of the colon is then performed as described above. Rectal resection is done in the TME (total mesorectal excision) plane with monopolar electrocoagulation, only laterally the distal dissection is carried out more proximal to the rectum to minimize nerve damage. The rectum is transected at the dentate line with a PI-30 stapling advice. The abdomen is closed with two running sutures (slowly absorbable monofilament suture).

#### Laparoscopic approach: standardised technique (Figure 1)

The pneumoperitoneum pressure is restricted to 14 mmHg. One monitor is placed near the left shoulder of the patient, another monitor between the legs of the patient. The operating surgeon stands between the legs in the beginning (mobilization of ascending colon and right flexure) and then on the right side of the patient for most of the remaining procedure. Dissection is predominantly conducted with ultrasonic shears or with Ligasure^®^. Laparoscopic colonic and rectal mobilization is done with a five trocar technique similar to the techniques already described (2, 10). The incisions are later incorporated into the periumbilical incision and used for the placement of drains and the fashioning of the ileostomy. First, ascending and descending colon including both flexures but excluding the greater omentum are mobilized. The rectum is then resected in the mesorectal plane down to the upper anal canal (TME) and finally transected at the anorectal junction using an Endo-GIA. A small periumbilical incision (< 4 cm) incorporating the camera port is then done and the large bowel eviscerated and dissected. The dissection of the colon can also be done intracorporeally. The ileoanal pouch is constructed extracorporally and the pouchanal anastomosis fashioned using a double stapling technique under camera vision.

Conversion from laparoscopic to a conventional approach is permitted when deemed necessary by the operating surgeons. The reason for conversion needs to be documented and the patient will remain in the laparoscopic group for statistical analysis according to the intention-to-treat principle.

### Sample size estimation and statistical considerations

Analysis of previous data from our department shows a mean difference in blood loss of 300 ml between the two groups. Assuming standard deviations of 300 and 600 65 patients per group are required to obtain a power of 80% (β = 0.2) with a significance level of 0,025 using a simple one-sided t-test for two groups. Assuming drop outs, 80 patients per group are required.

Statistical methods are used to assess the quality of data, homogeneity of treatment groups, endpoints and safety of the laparoscopic compared to the conventional procedure. The analysis is performed on the basis of an intention to treat (ITT) population and with respect to ITT principles. A patient belongs to the ITT population after the randomisation. The statistical analysis will be done using two one-sided t-tests at the 0,025 level to test superiority but also a possible inferiority of the laparoscopic versus the conventional technique. For the comparison of blood loss distributions the Mann-Whitney test will be used, for the comparison of proportions a chi-square test with continuity correction will be used. Corresponding effects (difference in location and odds ratio) will be estimated and reported together with their 95% confidence intervals. The analysis of secondary endpoints will be descriptive. The description of continuous variables includes at least numbers of observations, mean, standard deviation, median, minimum and maximum in the trial population. The description of nominal variables includes at least the number and percentage of patients belonging to the relevant categories. An interim analysis is not planned.

The ITT as well as per protocol analysis must be significant to get an overall significant result. If only one of these analyses is significant, the incompatibility must be discussed.

### Randomisation and blinding

A block-randomisation-list is generated via computer system (SAS Version 8.2., SAS Institute Inc., Cary, USA). The sealed randomisation list is stored in the investigator file. For each enrolled patient there is an opaque envelope available. Randomisation is done on the day of surgery, at least one hour before the procedure in order to allow adequate preparation, i.e. OR equipment. Patients and pain assessor are not informed on the type of operation performed. Randomisation is stratified according to familial polyposis or ulcerative colitis. The patient and the pain assessor are blinded for the technique used in order to prevent an influence on postoperative pain and the post-surgical ranking until day two after surgery. Postoperatively in the operating theatre there will be a dressing all over the abdomen, completely covering all possible incisions. The first change of the dressing postoperatively will be done after assessing the secondary endpoints on the second postoperative day. If required, e.g. contamination of the dressing, the dressing is changed before the second postoperative day, with a blindfolded patient. If a patient does not consent to being blindfolded he will remain in the study for the primary endpoint but he will be excluded in the analysis of postoperative pain and post-surgical ranking.

### Investigators and safety aspects

Patients will be recruited by the Department of Surgery at the University of Heidelberg. The Department is a nation-wide referral centre for the treatment of chronic inflammatory bowel diseases and familial polyposis. Minimal invasive techniques have been used for performing restorative proctocolectomy from 1999 onwards. In 2001 a completely laparoscopic technique was introduced. As only very few centres in Germany and world-wide have a broad experience in minimal invasive surgery for performing restorative proctocolectomy, this trial was planed as a single centre, expertise based study. All investigators performing the surgery are hospital-based visceral surgeons with a broad experience in minimal invasive surgery. As the performance of the procedure is dependent on the experience of the surgeon, only surgeons who have operated at least 10 laparoscopic, 10 conventional restorative proctocolectomies and who have additionally assisted more than 10 cases of each procedure, can participate in this study. To minimise bias a standardised procedure is performed in both groups according to a treatment manual and special training within the department.

All serious adverse events (SAE) will be reported to the principal investigator and the leading ethics committee. For the safety analysis the incidence of adverse events (AE) and SAE will be analyzed. Patients may at any time withdraw from the study either at their own request or at the request of the principal investigator.

### Ethics and informed consent

The final protocol was approved by the ethics committee of the University of Heidelberg, Medical School. Written informed consent is obtained from each patient in oral and written form before inclusion in the trial and the nature, scope and possible consequences of the trial have been explained by a physician. The investigator will not undertake any measures specifically required only for the clinical trial until valid consent has been obtained.

Two amendments were accepted before this publication by the ethical committees:

The reason for the first amendment was to adjust one exclusion criteria (malignant disease and high grade dysplasia). Due to the results of recently published large randomized studies the laparoscopic approach is now generally accepted as a safe alternative to open surgery in colorectal cancer in terms of morbidity, hospital stay, tumor recurrence and cancer-related survival. Therefore, the exclusion criteria "malignant dieseas and high grade dysplasia" for patients in this trial was removed and patients with non-advanced tumors (≤T2) from then on included.

The reason for the second amendment was to expand the assessment of one of the secondary endpoints, i.e. postoperative pain. This was previously not subdivided into pain at rest and in motion, in the amendment this is now included.

### Follow up

The follow-up will be continued for up to 12 months after primary surgery. An interview by phone will be held 3, 6 and 12 months post operation and implies past medical history, personal data, basic study related examination and physical examination. The first visits on ward are made on day 1 post surgery, day 2 and day of discharge.

### Data management and quality assurance

Investigators enter data directly in a paper-based case report form (CRF). These are arranged for each visit time-point and contain instructions and relevant definitions. All treatments are recorded in treatment logs. Standard adverse events forms are used to document (serious) adverse events and relevant clinical procedures that have been carried out. The principle investigator is responsible for the documentation and reporting of serious adverse events to the independent ethical committee. Serious adverse events have to be reported to the principle investigator within 24 hours or not later than the next working day.

### Current status and planning

The duration of this clinical trial is expected to be 4 years, with subject enrolment starting in September 2004. About 2–4 patients per month can be enrolled. Evaluation and reporting of the clinical results will be done within three months after closing the clinical database, which will be two months after the end of the recruitment. The follow up database will be closed 2 months after the last follow up. Up to April 2006, a total of 25 patients were enrolled; 13 underwent the conventional, 12 the laparoscopic approach. A total of 62 patients fulfilling the inclusion criteria were screened in the same time period.

## Discussion

Publishing study protocols is increasingly being done, also in surgery [18,19]. The aim is to improve transparency in clinical trials and thereby also contribute to alleviating the problem of publication bias. Furthermore, providing detailed descriptions of the methodology of a clinical trial to the scientific society prior to its conduction will help to increase reliability and validity of the results and findings as the adherence to the guidelines of good clinical practice can be adequately analysed. Finally, many details of a study design cannot be published in a results paper but these details may be of profound interest to researchers wanting to perform similar studies.

The primary objective of the LapConPouch-Study is to compare a totally laparoscopic with the standard conventional approach in regard to intraoperative blood loss and need for perioperative blood transfusions. We hypothesise that intraoperative blood loss and the need for preoperative blood transfusions are significantly higher in the conventional group. There is controversy in the literature on the actual benefit of minimal invasive techniques for extensive colorectal procedures. Up until now, only one prospective randomised clinical trial that compares conventional restorative proctocolectomy to a minimal invasive approach, in this case to a laparoscopically assisted technique has been published in the literature [14]. This study could not find any advantage for the minimally invasive procedure in regard to postoperative quality of life. One may argue that the choice of estimated blood loss is not very objective or quantitative. Obviously, blood loss is a surrogate end point in this context. It would be preferable to use major complications as primary end point but when calculating the necessary sample size (at least 300 patients per group) and considering the paucity of this operation overall such a study would not be realistic and could not be done. In a recent published meta-analysis [21] on "short-term outcomes of laparoscopic versus open approaches to ileal pouch surgery" blood loss was estimated to be 84 ml less in the minimal invasive group supporting the results from our own series and the use of blood loss as a surrogate end point. In order to minimize bias the blood loss has to be independently estimated by the anesthesiologist, the surgeon and the scrub nurse. Moreover, the blood collected in the suction is measured (minus the rinsing fluid, if any has been used). We feel that these methods adequately minimize bias in regard to blood loss.

The number of blood units given perioperatively as the other primary endpoint is obviously dependant on the policy of blood transfusion and therefore also susceptible to bias. The policy of our department has become very restricted and standardized over the last 5 years. Patients are only transfused if the hemoglobin falls below 6 mg% (in known cardiac disease under 9 mg%) or if they become unstable. These rules are strictly adhered to, somewhat minimizing subjectiveness in this regard.

Another critical aspect of our study design is that the surgeons, in knowledge of the endpoint, may thus be biased in the method of conduct of surgery (conscious or unconscious). In principle, this assumption is correct but the strict requirement of blinding the surgeon in regard to the endpoint would virtually render most surgical trials for less common indications impossible. In the case of laparoscopic restorative proctocolectomy, the number of surgeons performing this procedure is limited and obviously these surgeons know the potential advantages and disadvantages of the different surgical techniques and cannot be blinded to the procedure they are performing.

The laparoscopically assisted technique was more costly than the open procedure. However, laparoscopic purists could argue that the laparoscopically assisted technique with a Pfannenstiel incision used in this trial reduced the potential benefit of a truly minimally invasive procedure thereby resulting in the differences failing statistical significance. A totally laparoscopic technique has not yet been investigated in a randomized trial thus justifying the execution of the LapConPouch study. Currently the conventional approach to restorative proctocolectomy is the gold standard; however, specialized centers are increasingly performing this operation laparoscopically. Patients are often told that the minimally invasive approach carries several clear advantages but these have not yet been adequately demonstrated in randomized controlled trials. In the contrary, at best equivalence has been shown in regard to complications and quality of life. After the learning curve for minimal invasive restorative proctocolectomy has been overcome in the specialized centers, a randomized study is now feasible and necessary in order to define the future role of the new approach.

One of the major problems encountered in the trial so far was the refusal of more than half of the potentially eligible patients to take part in the study. The majority of these patients had come with the explicit desire to have a laparoscopic procedure. These patients were often extremely well informed and had chosen our department due to our large published expertise in the field of minimally invasive restorative proctocolectomy. Even, after intensive informed consent on the current available data and the goal of this study, most of these patients were not prepared to change their decision for a laparoscopic procedure. In this context, a multicenter study would obviously be preferable in order to better achieve the calculated patient numbers, but this is not realistic because only very few centers worldwide perform laparoscopic restorative proctocolectomy with the totally laparoscopic technique as described above.

In statistical regard, a two tailed approach would obviously be the better choice but would also substantially increase the needed sample size resulting in the already discussed problems of inadequate patient recruitment. In the clinical setting one-sided statistical analysis are generally being more accepted albeit their known drawbacks. We argue that in this specific situation a one-sided approach is acceptable. One of the main prerequisites in this setting is the prior specification of the one-sided analysis which is abided to in this protocol. As to the expected association of blood loss and complications, the data of our study will hopefully help to clarify this issue.

The design of this single center RCT will allow the generation of results with adequate statistical power and internal validity. The results could potentially have a relevant impact on the future use of minimal invasive techniques for performing this and also other complex colorectal operation. If the results can show a significant reduction of blood loss and a lower rate of blood transfusions, patients will have a potential benefit in terms of less risk of overall complications and less infectious risk as these are known to be correlated to larger blood loss and transfusions [20]. The conduction of this study will contribute to implementing the practices of "Evidence-based Medicine" (EBM) in surgery and hopefully help to clarify the role of laparoscopic restorative proctocolectomy.

## Competing interests

The author(s) declare that they have no competing interests.

## Authors' contributions

PK, HPK, JW, MWB and CMS designed the study and contributed to manuscript preparation. JS, CNG, MK and DA conduct the study. DA and PK drafted the manuscript. All authors read and approved the final manuscript.

## Pre-publication history

The pre-publication history for this paper can be accessed here:



## Figures and Tables

**Figure 1:**
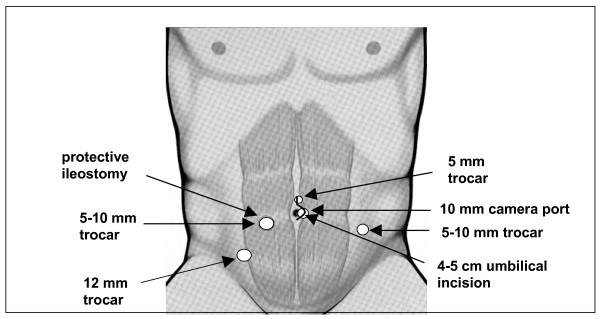
Positioning of trocars, incision and ileostomy for the totally laparoscopic technique.

**Table 1:** Eligibility Criteria

**Inclusion Criteria**
At study enrolment
• Age equal or greater than 14 years (providing informed consent by patient and by parents or legal guardian between 16–18, only patient when 18 or older)
• Patients with benign disease (familial polyposis or ulcerative colitis) scheduled for elective surgery
• Patient or guardian has given informed consent
• Both standardised surgical approaches are suitable for treatment
At the end of surgical exploration
• Suitable for restorative proctocolectomy
**Exclusion Criteria**
• Active malignant disease or high suspicion for malignancy (clinical and imaging evidence, e.g. higher grade dysplasia on histology) *
• Previous median laparotomy or Pfannenstiel incision (excluding limited incision such as appendectomy, cholecystectomy etc.)
• Participation in another intervention-trial with interference of intervention and outcome
• Severe psychiatric or neurologic diseases
• Drug- and/or alcohol-abuse according to local standards
• Coagulopathy

* modified to patients with non-advanced tumors (≤T2), see subheading ethics and informed consent
